# *rac*-Ethyl 2-hy­droxy-2,7,7-trimethyl-4-(4-nitro­phen­yl)-5-oxo-3,4,5,6,7,8-hexa­hydro-2*H*-chromene-3-carboxyl­ate

**DOI:** 10.1107/S1600536812050581

**Published:** 2012-12-15

**Authors:** Abel M. Maharramov, Arif I. Ismiev, Bahruz A. Rashidov, Rizvan K. Askerov, Konstantin A. Potekhin

**Affiliations:** a Baku State University, Baku, Z. Khalilov St 23, AZ-1148, Azerbaijan; b Vladimir State University, 600000 Vladimir, Gor’ky St 87, Russia

## Abstract

The title mol­ecule, C_21_H_25_NO_7_, has four stereogenic centres and crystallized as a racemate. It consists of enanti­omeric pairs with the relative configuration *rac*-(1R*,2S*,3R*). The cyclo­hexenone ring adopts an envelope conformation; the dimethyl-substituted C atom lies 0.640 (1) Å out of the mean plane formed by the rest of the ring atoms (r.m.s. deviation = 0.016 Å). The oxacyclo­hexene ring adopts a half-chair conformation, the hy­droxy- and carboxyl-substituted C atoms lying −0.336 (1) and 0.419 (1) Å, respectively, out of the mean plane formed by the rest of the ring atoms (r.m.s. deviation = 0.002 Å). In the crystal, O—H⋯O hydrogen bonds link the mol­ecules into a chain along the *c-*axis direction.

## Related literature

For general background to 2*H*-chromenes and their derivatives, see: Cai (2008 ▶); Valenti *et al.* (1993 ▶); Hyana & Saimoto (1987 ▶); Tang *et al.* (2007 ▶). For their anti­cancer activity, see: Afanti­tis *et al.* (2006 ▶); Cai (2007 ▶). For puckering parameters, see: Cremer & Pople (1975 ▶).
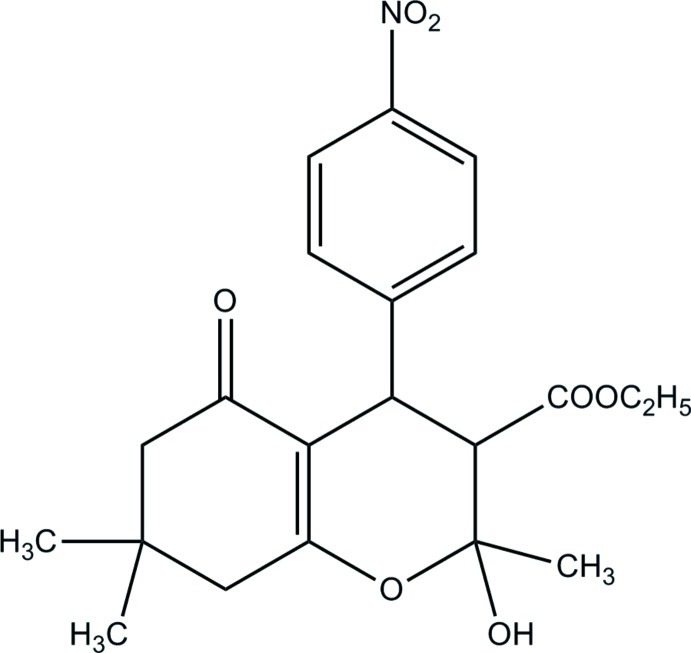


## Experimental

### 

#### Crystal data

C_21_H_25_NO_7_
*M* *_r_* = 403.42
Monoclinic, 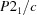

*a* = 10.4163 (8) Å
*b* = 24.2608 (18) Å
*c* = 8.0796 (6) Å
β = 96.437 (2)°
*V* = 2028.9 (3) Å^3^
*Z* = 4
Mo *K*α radiation
μ = 0.10 mm^−1^
*T* = 296 K
0.30 × 0.20 × 0.20 mm

#### Data collection

Bruker APEXII CCD diffractometer
Absorption correction: multi-scan (*SADABS*; Sheldrick, 1998 ▶) *T*_min_ = 0.971, *T*_max_ = 0.980
23423 measured reflections
5078 independent reflections
4084 reflections with *I* > 2σ(*I*)
*R*_int_ = 0.022

#### Refinement

*R*[*F* ^2^ > 2σ(*F* ^2^)] = 0.044
*wR*(*F* ^2^) = 0.118
*S* = 1.00
5078 reflections
266 parameters
H atoms treated by a mixture of independent and constrained refinement
Δρ_max_ = 0.30 e Å^−3^
Δρ_min_ = −0.20 e Å^−3^

### 

Data collection: *APEX2* (Bruker, 2005 ▶); cell refinement: *SAINT-Plus* (Bruker, 2005 ▶); data reduction: *SAINT-Plus*; program(s) used to solve structure: *SHELXTL* (Sheldrick, 2008 ▶); program(s) used to refine structure: *SHELXTL*; molecular graphics: *SHELXTL*; software used to prepare material for publication: *SHELXTL*.

## Supplementary Material

Crystal structure: contains datablock(s) global, I. DOI: 10.1107/S1600536812050581/aa2078sup1.cif

Structure factors: contains datablock(s) I. DOI: 10.1107/S1600536812050581/aa2078Isup2.hkl

Supplementary material file. DOI: 10.1107/S1600536812050581/aa2078Isup3.cml

Additional supplementary materials:  crystallographic information; 3D view; checkCIF report

## Figures and Tables

**Table 1:** Hydrogen-bond geometry (Å, °)

*D*—H⋯*A*	*D*—H	H⋯*A*	*D*⋯*A*	*D*—H⋯*A*
O2—H2⋯O3^i^	0.82 (2)	2.08 (2)	2.8881 (15)	172 (2)

Symmetry code: (i) 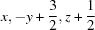.

**Fig. 1.**
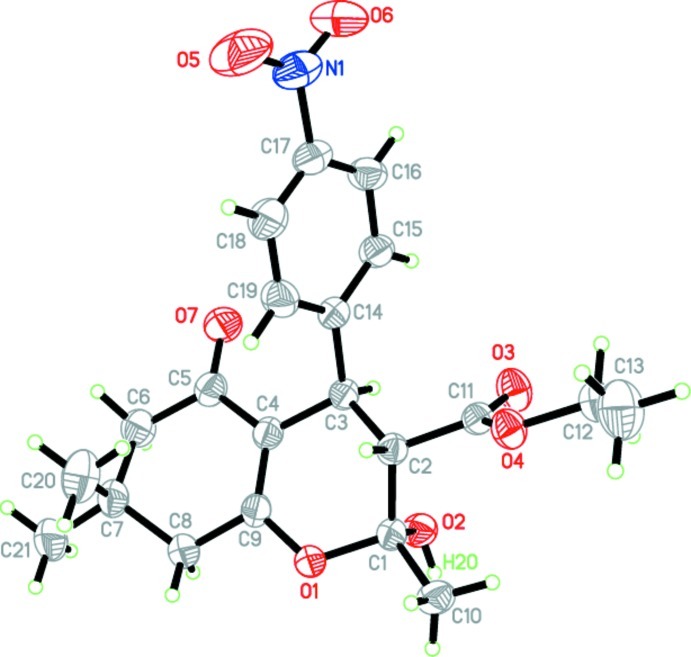
The molecular structure of the title compound, with the atomic numbering scheme. Displacement ellipsoids were drawn at the 50% probability level.

**Fig. 2.**
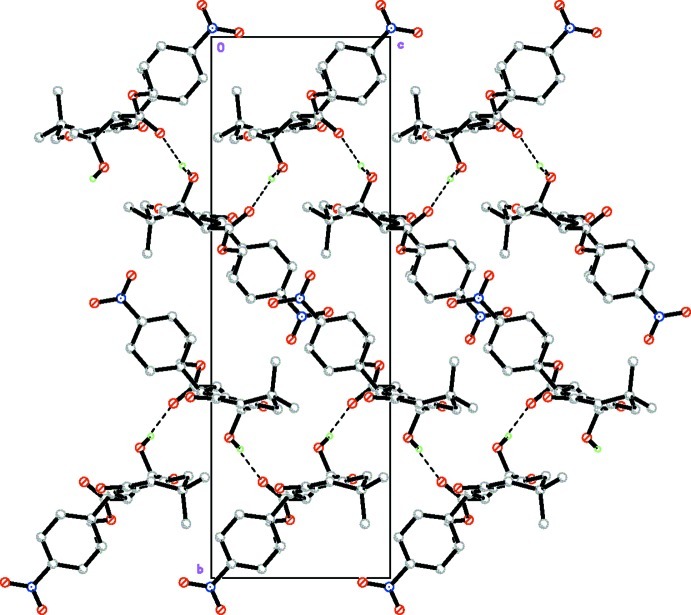
The hydrogen-bonded (dashed lines) packing in the title compound (a view perpendicular to the bc plane); H-atoms not involved in hydrogen bonding are omitted for clarity.

## supplementary crystallographic information

### Comment

2*H*-chromenes and their derivatives possess various biological and pharmacological properties such as anti-viral, anti-fungal, anti-inflammatory, antidiabetic, cardionthonic, anti-anaphylactic and anti-cancer activity (Cai, 2008; Valenti *et al.*, 1993; Hyana & Saimoto, 1987; Tang *et al.*, 2007). 2*H*-chromenes are a new series of apoptosis inducers, which exhibit potent anticancer activity (Afantitis *et al.*, 2006; Cai, 2007;). Considering the importance of 2*H*-chromene-3-carboxylate derivatives, a single-crystal X-ray diffraction study on the title compound was carried out and analyzed.

A simple synthesis of new and biologically active ethyl 2-hydroxy-2,7,7-trimethyl-4-(4-nitrophenyl)-5-oxo-3,4,5,6,7,8-hexahydro-2*H*- chromene-3-carboxylate (Fig. 1) was carried out using cyclocondensation reaction of 4-nitrobenzaldehyde, acetoacetic ether and dimedone with allylamine as catalizator at room temperature. The title molecule is chiral with four stereogenic centres. The crystal is a racemate and consists of enantiomeric pairs with the relative configuration *rac*-(*1R*, 2S*, 3R**). The cyclohexenone ring adopt a envelope conformation, C7 lies 0.640 (1) Å out of the plane formed by the rest of the ring atoms (r.m.s. deviation = 0.016 Å). The oxacyclohexene ring adopt a half-chair conformation, C1 and C2 lie -0.336 (1) and 0.419 (1) Å respectively out of the plane formed by the rest of the ring (r.m.s. deviation = 0.002 Å). The 4-nitrophenyl ring is in a pseudo-equatorial position. The torsion angle between the ethoxycarbonyl group and the 4-nirtophenyl substituent C11–C2–C3–C14 is -65.16 (13) ° that indicates the pseudo-axial location of hydrogen atoms at C2 and C3. Intermolecular O—H···O hydrogen bonds link molecules into a chain along the *c* axis (Table 1, Fig. 2).

### Experimental

Dimedone (5 mmol), 4-nitrobenzaldehyde (5 mmol) and acetoacetic ether (5 mmol) were dissolved in ethanol (15 ml). Then allylamine (0.4 ml) was added and mixture was stirred at 300 K for 3 h. Obtained yellow crystals were filtered and washed with ethanol. The crystals were dissolved in ethanol (20 ml) and recrystallized to yield yellow crystals of the title compound suitable for X-ray analysis.

### Refinement

Hydrogen atom of the OH group was found in difference-Fourier maps and included in the refinement with isotropic displacement parameter. The other hydrogen atoms were placed in calculated positions and refined in the riding mode with fixed isotropic displacement parameters [*U*_iso_(H) = 1.2*U*_eq_(C) or 1.5*U*_eq_(C)].

### Crystal data

**Table:**

C_21_H_25_NO_7_	*F*(000) = 856
*M**_r_* = 403.42	*D*_x_ = 1.321 Mg m^−^^3^
Monoclinic, *P*2_1_/*c*	Mo *K*α radiation, λ = 0.71073 Å
Hall symbol: -P 2ybc	Cell parameters from 6760 reflections
*a* = 10.4163 (8) Å	θ = 2.7–28.5°
*b* = 24.2608 (18) Å	µ = 0.10 mm^−^^1^
*c* = 8.0796 (6) Å	*T* = 296 K
β = 96.437 (2)°	Prism, yellow
*V* = 2028.9 (3) Å^3^	0.30 × 0.20 × 0.20 mm
*Z* = 4	

### Data collection

**Table:**

Bruker APEXII CCD diffractometer	5078 independent reflections
Radiation source: fine-focus sealed tube	4084 reflections with *I* > 2σ(*I*)
Graphite monochromator	*R*_int_ = 0.022
φ and ω scans	θ_max_ = 28.4°, θ_min_ = 1.7°
Absorption correction: multi-scan (*SADABS*; Sheldrick, 1998)	*h* = −13→13
*T*_min_ = 0.971, *T*_max_ = 0.980	*k* = −32→32
23423 measured reflections	*l* = −10→10

### Refinement

**Table:**

Refinement on *F*^2^	Primary atom site location: structure-invariant direct methods
Least-squares matrix: full	Secondary atom site location: difference Fourier map
*R*[*F*^2^ > 2σ(*F*^2^)] = 0.044	Hydrogen site location: difference Fourier map
*wR*(*F*^2^) = 0.118	H atoms treated by a mixture of independent and constrained refinement
*S* = 1.00	*w* = 1/[σ^2^(*F*_o_^2^) + (0.0521*P*)^2^ + 0.7463*P*] where *P* = (*F*_o_^2^ + 2*F*_c_^2^)/3
5078 reflections	(Δ/σ)_max_ = 0.001
266 parameters	Δρ_max_ = 0.30 e Å^−^^3^
0 restraints	Δρ_min_ = −0.20 e Å^−^^3^

### Special details

**Table:**

Geometry. All s.u.'s (except the s.u. in the dihedral angle between two l.s. planes) are estimated using the full covariance matrix. The cell s.u.'s are taken into account individually in the estimation of s.u.'s in distances, angles and torsion angles; correlations between s.u.'s in cell parameters are only used when they are defined by crystal symmetry. An approximate (isotropic) treatment of cell s.u.'s is used for estimating s.u.'s involving l.s. planes.
Refinement. Refinement of *F*^2^ against ALL reflections. The weighted *R*-factor *wR* and goodness of fit *S* are based on *F*^2^, conventional *R*-factors *R* are based on *F*, with *F* set to zero for negative *F*^2^. The threshold expression of *F*^2^ > σ(*F*^2^) is used only for calculating *R*-factors(gt) *etc*. and is not relevant to the choice of reflections for refinement. *R*-factors based on *F*^2^ are statistically about twice as large as those based on *F*, and *R*- factors based on ALL data will be even larger.

### Fractional atomic coordinates and isotropic or equivalent isotropic displacement parameters (Å^2^)

**Table:**

	*x*	*y*	*z*	*U*_iso_*/*U*_eq_	
O1	0.06693 (9)	0.68270 (4)	0.29152 (11)	0.0360 (2)	
O2	−0.04636 (10)	0.74080 (4)	0.10075 (13)	0.0383 (2)	
H2	−0.080 (2)	0.7620 (9)	0.162 (3)	0.061 (6)*	
O3	−0.17047 (10)	0.67735 (4)	−0.21204 (13)	0.0427 (3)	
O4	−0.23488 (9)	0.60735 (4)	−0.06145 (12)	0.0366 (2)	
O5	0.23965 (18)	0.44094 (6)	−0.4474 (2)	0.0829 (5)	
O6	0.16430 (16)	0.49162 (6)	−0.65423 (17)	0.0721 (4)	
O7	0.35300 (11)	0.66576 (5)	−0.11760 (13)	0.0508 (3)	
N1	0.18908 (15)	0.48317 (6)	−0.5053 (2)	0.0522 (4)	
C1	−0.04561 (12)	0.68823 (6)	0.16883 (16)	0.0314 (3)	
C2	−0.03255 (12)	0.64615 (5)	0.03021 (15)	0.0297 (3)	
H2A	−0.0244	0.6095	0.0812	0.036*	
C3	0.08949 (12)	0.65704 (5)	−0.05552 (16)	0.0303 (3)	
H3A	0.0758	0.6908	−0.1218	0.036*	
C4	0.20193 (13)	0.66649 (6)	0.07676 (16)	0.0322 (3)	
C5	0.33285 (14)	0.66857 (6)	0.02822 (18)	0.0370 (3)	
C6	0.44363 (14)	0.67805 (8)	0.1622 (2)	0.0467 (4)	
H6A	0.4666	0.7168	0.1616	0.056*	
H6B	0.5176	0.6573	0.1335	0.056*	
C7	0.41940 (14)	0.66238 (7)	0.33864 (19)	0.0411 (3)	
C8	0.29096 (13)	0.68842 (7)	0.37109 (18)	0.0393 (3)	
H8A	0.2660	0.6740	0.4748	0.047*	
H8B	0.3028	0.7279	0.3842	0.047*	
C9	0.18422 (12)	0.67797 (5)	0.23553 (17)	0.0323 (3)	
C10	−0.15932 (14)	0.67843 (7)	0.26566 (19)	0.0432 (3)	
H10A	−0.1609	0.7063	0.3498	0.065*	
H10B	−0.1514	0.6428	0.3174	0.065*	
H10C	−0.2379	0.6800	0.1914	0.065*	
C11	−0.15205 (12)	0.64616 (5)	−0.09602 (16)	0.0310 (3)	
C12	−0.35486 (14)	0.60395 (7)	−0.17297 (19)	0.0407 (3)	
H12A	−0.3363	0.6005	−0.2875	0.049*	
H12B	−0.4062	0.6369	−0.1634	0.049*	
C13	−0.42646 (19)	0.55435 (8)	−0.1231 (3)	0.0638 (5)	
H13A	−0.5062	0.5509	−0.1944	0.096*	
H13B	−0.4443	0.5583	−0.0097	0.096*	
H13C	−0.3748	0.5220	−0.1333	0.096*	
C14	0.11142 (13)	0.60989 (6)	−0.17235 (16)	0.0325 (3)	
C15	0.08635 (14)	0.61724 (6)	−0.34299 (17)	0.0371 (3)	
H15A	0.0540	0.6508	−0.3847	0.044*	
C16	0.10879 (15)	0.57534 (6)	−0.45185 (18)	0.0410 (3)	
H16A	0.0915	0.5804	−0.5663	0.049*	
C17	0.15694 (14)	0.52615 (6)	−0.38837 (19)	0.0396 (3)	
C18	0.17904 (16)	0.51659 (6)	−0.2196 (2)	0.0456 (4)	
H18A	0.2094	0.4826	−0.1788	0.055*	
C19	0.15505 (16)	0.55868 (6)	−0.11237 (18)	0.0428 (3)	
H19A	0.1683	0.5527	0.0020	0.051*	
C20	0.41427 (19)	0.59983 (8)	0.3586 (3)	0.0623 (5)	
H20A	0.4956	0.5841	0.3380	0.093*	
H20B	0.3467	0.5850	0.2806	0.093*	
H20C	0.3973	0.5910	0.4699	0.093*	
C21	0.52760 (16)	0.68578 (9)	0.4625 (2)	0.0565 (5)	
H21A	0.6084	0.6694	0.4427	0.085*	
H21B	0.5101	0.6776	0.5740	0.085*	
H21C	0.5322	0.7250	0.4485	0.085*	

### Atomic displacement parameters (Å^2^)

**Table:**

	*U*^11^	*U*^22^	*U*^33^	*U*^12^	*U*^13^	*U*^23^
O1	0.0287 (5)	0.0509 (6)	0.0282 (4)	0.0038 (4)	0.0028 (4)	−0.0004 (4)
O2	0.0428 (6)	0.0338 (5)	0.0386 (5)	0.0051 (4)	0.0056 (4)	−0.0010 (4)
O3	0.0407 (6)	0.0451 (6)	0.0406 (5)	−0.0048 (4)	−0.0030 (4)	0.0116 (4)
O4	0.0338 (5)	0.0386 (5)	0.0367 (5)	−0.0063 (4)	−0.0001 (4)	0.0030 (4)
O5	0.1139 (14)	0.0500 (8)	0.0884 (11)	0.0253 (8)	0.0266 (10)	−0.0098 (7)
O6	0.0927 (11)	0.0727 (9)	0.0532 (8)	0.0037 (8)	0.0182 (7)	−0.0222 (7)
O7	0.0390 (6)	0.0750 (8)	0.0403 (6)	0.0008 (5)	0.0130 (5)	−0.0018 (5)
N1	0.0546 (8)	0.0424 (8)	0.0626 (9)	−0.0018 (6)	0.0190 (7)	−0.0137 (7)
C1	0.0268 (6)	0.0373 (7)	0.0303 (6)	0.0010 (5)	0.0037 (5)	0.0006 (5)
C2	0.0287 (6)	0.0310 (6)	0.0296 (6)	0.0016 (5)	0.0035 (5)	0.0019 (5)
C3	0.0291 (6)	0.0326 (6)	0.0293 (6)	0.0035 (5)	0.0043 (5)	0.0014 (5)
C4	0.0277 (6)	0.0348 (7)	0.0341 (6)	0.0019 (5)	0.0036 (5)	0.0006 (5)
C5	0.0322 (7)	0.0394 (7)	0.0399 (7)	0.0011 (5)	0.0065 (6)	0.0009 (6)
C6	0.0292 (7)	0.0652 (10)	0.0461 (8)	−0.0052 (7)	0.0053 (6)	−0.0018 (7)
C7	0.0297 (7)	0.0498 (9)	0.0429 (8)	0.0030 (6)	0.0002 (6)	0.0019 (6)
C8	0.0324 (7)	0.0495 (8)	0.0349 (7)	0.0022 (6)	−0.0005 (5)	−0.0021 (6)
C9	0.0277 (6)	0.0341 (7)	0.0348 (7)	0.0029 (5)	0.0027 (5)	0.0024 (5)
C10	0.0341 (7)	0.0583 (9)	0.0389 (8)	−0.0044 (6)	0.0111 (6)	−0.0066 (7)
C11	0.0301 (6)	0.0311 (6)	0.0322 (6)	0.0009 (5)	0.0050 (5)	−0.0018 (5)
C12	0.0315 (7)	0.0476 (8)	0.0418 (7)	−0.0025 (6)	−0.0008 (6)	−0.0028 (6)
C13	0.0486 (10)	0.0582 (11)	0.0821 (13)	−0.0178 (8)	−0.0033 (9)	0.0004 (10)
C14	0.0294 (6)	0.0362 (7)	0.0321 (6)	0.0027 (5)	0.0047 (5)	−0.0005 (5)
C15	0.0412 (7)	0.0371 (7)	0.0336 (7)	0.0074 (6)	0.0073 (6)	0.0021 (5)
C16	0.0461 (8)	0.0454 (8)	0.0324 (7)	0.0014 (6)	0.0087 (6)	−0.0019 (6)
C17	0.0379 (7)	0.0371 (7)	0.0453 (8)	−0.0003 (6)	0.0112 (6)	−0.0077 (6)
C18	0.0509 (9)	0.0348 (7)	0.0509 (9)	0.0091 (6)	0.0041 (7)	0.0015 (6)
C19	0.0517 (9)	0.0407 (8)	0.0349 (7)	0.0074 (7)	0.0001 (6)	0.0028 (6)
C20	0.0519 (10)	0.0541 (11)	0.0790 (13)	0.0110 (8)	−0.0003 (9)	0.0108 (9)
C21	0.0344 (8)	0.0817 (13)	0.0508 (9)	0.0015 (8)	−0.0066 (7)	−0.0023 (9)

### Geometric parameters (Å, º)

**Table:**

O1—C9	1.3544 (16)	C8—C9	1.4924 (18)
O1—C1	1.4541 (15)	C8—H8A	0.9700
O2—C1	1.3885 (17)	C8—H8B	0.9700
O2—H2	0.82 (2)	C10—H10A	0.9600
O3—C11	1.2032 (16)	C10—H10B	0.9600
O4—C11	1.3278 (16)	C10—H10C	0.9600
O4—C12	1.4588 (17)	C12—C13	1.494 (2)
O5—N1	1.221 (2)	C12—H12A	0.9700
O6—N1	1.219 (2)	C12—H12B	0.9700
O7—C5	1.2216 (17)	C13—H13A	0.9600
N1—C17	1.4706 (19)	C13—H13B	0.9600
C1—C10	1.5096 (19)	C13—H13C	0.9600
C1—C2	1.5328 (18)	C14—C15	1.3857 (19)
C2—C11	1.5182 (18)	C14—C19	1.391 (2)
C2—C3	1.5373 (18)	C15—C16	1.381 (2)
C2—H2A	0.9800	C15—H15A	0.9300
C3—C4	1.5119 (18)	C16—C17	1.372 (2)
C3—C14	1.5166 (18)	C16—H16A	0.9300
C3—H3A	0.9800	C17—C18	1.377 (2)
C4—C9	1.3454 (19)	C18—C19	1.380 (2)
C4—C5	1.4615 (19)	C18—H18A	0.9300
C5—C6	1.509 (2)	C19—H19A	0.9300
C6—C7	1.524 (2)	C20—H20A	0.9600
C6—H6A	0.9700	C20—H20B	0.9600
C6—H6B	0.9700	C20—H20C	0.9600
C7—C20	1.528 (2)	C21—H21A	0.9600
C7—C8	1.529 (2)	C21—H21B	0.9600
C7—C21	1.530 (2)	C21—H21C	0.9600
			
C9—O1—C1	117.94 (10)	C1—C10—H10A	109.5
C1—O2—H2	108.7 (15)	C1—C10—H10B	109.5
C11—O4—C12	116.24 (11)	H10A—C10—H10B	109.5
O6—N1—O5	123.61 (15)	C1—C10—H10C	109.5
O6—N1—C17	118.46 (15)	H10A—C10—H10C	109.5
O5—N1—C17	117.93 (15)	H10B—C10—H10C	109.5
O2—C1—O1	108.85 (11)	O3—C11—O4	124.00 (12)
O2—C1—C10	112.34 (12)	O3—C11—C2	124.80 (12)
O1—C1—C10	104.57 (11)	O4—C11—C2	111.20 (11)
O2—C1—C2	108.62 (10)	O4—C12—C13	107.34 (13)
O1—C1—C2	107.81 (10)	O4—C12—H12A	110.2
C10—C1—C2	114.38 (11)	C13—C12—H12A	110.2
C11—C2—C1	110.94 (10)	O4—C12—H12B	110.2
C11—C2—C3	110.76 (10)	C13—C12—H12B	110.2
C1—C2—C3	111.12 (11)	H12A—C12—H12B	108.5
C11—C2—H2A	108.0	C12—C13—H13A	109.5
C1—C2—H2A	108.0	C12—C13—H13B	109.5
C3—C2—H2A	108.0	H13A—C13—H13B	109.5
C4—C3—C14	113.29 (11)	C12—C13—H13C	109.5
C4—C3—C2	108.77 (10)	H13A—C13—H13C	109.5
C14—C3—C2	109.91 (11)	H13B—C13—H13C	109.5
C4—C3—H3A	108.2	C15—C14—C19	118.63 (13)
C14—C3—H3A	108.2	C15—C14—C3	119.84 (12)
C2—C3—H3A	108.2	C19—C14—C3	121.53 (12)
C9—C4—C5	118.72 (12)	C16—C15—C14	120.83 (13)
C9—C4—C3	121.82 (12)	C16—C15—H15A	119.6
C5—C4—C3	119.19 (12)	C14—C15—H15A	119.6
O7—C5—C4	121.55 (13)	C17—C16—C15	118.89 (13)
O7—C5—C6	120.01 (13)	C17—C16—H16A	120.6
C4—C5—C6	118.32 (12)	C15—C16—H16A	120.6
C5—C6—C7	116.08 (13)	C16—C17—C18	122.03 (13)
C5—C6—H6A	108.3	C16—C17—N1	118.47 (14)
C7—C6—H6A	108.3	C18—C17—N1	119.48 (14)
C5—C6—H6B	108.3	C17—C18—C19	118.37 (14)
C7—C6—H6B	108.3	C17—C18—H18A	120.8
H6A—C6—H6B	107.4	C19—C18—H18A	120.8
C6—C7—C20	111.00 (15)	C18—C19—C14	121.16 (14)
C6—C7—C8	107.29 (12)	C18—C19—H19A	119.4
C20—C7—C8	110.53 (14)	C14—C19—H19A	119.4
C6—C7—C21	109.40 (14)	C7—C20—H20A	109.5
C20—C7—C21	109.35 (14)	C7—C20—H20B	109.5
C8—C7—C21	109.22 (13)	H20A—C20—H20B	109.5
C9—C8—C7	113.47 (12)	C7—C20—H20C	109.5
C9—C8—H8A	108.9	H20A—C20—H20C	109.5
C7—C8—H8A	108.9	H20B—C20—H20C	109.5
C9—C8—H8B	108.9	C7—C21—H21A	109.5
C7—C8—H8B	108.9	C7—C21—H21B	109.5
H8A—C8—H8B	107.7	H21A—C21—H21B	109.5
C4—C9—O1	124.14 (12)	C7—C21—H21C	109.5
C4—C9—C8	124.36 (12)	H21A—C21—H21C	109.5
O1—C9—C8	111.48 (11)	H21B—C21—H21C	109.5
			
C9—O1—C1—O2	−73.27 (14)	C5—C4—C9—C8	−5.2 (2)
C9—O1—C1—C10	166.48 (12)	C3—C4—C9—C8	−179.15 (13)
C9—O1—C1—C2	44.38 (15)	C1—O1—C9—C4	−14.56 (19)
O2—C1—C2—C11	−66.83 (13)	C1—O1—C9—C8	164.06 (11)
O1—C1—C2—C11	175.37 (10)	C7—C8—C9—C4	−24.3 (2)
C10—C1—C2—C11	59.55 (15)	C7—C8—C9—O1	157.08 (12)
O2—C1—C2—C3	56.85 (13)	C12—O4—C11—O3	−0.54 (19)
O1—C1—C2—C3	−60.94 (13)	C12—O4—C11—C2	179.02 (11)
C10—C1—C2—C3	−176.77 (11)	C1—C2—C11—O3	84.53 (16)
C11—C2—C3—C4	170.27 (11)	C3—C2—C11—O3	−39.36 (18)
C1—C2—C3—C4	46.48 (14)	C1—C2—C11—O4	−95.03 (13)
C11—C2—C3—C14	−65.16 (13)	C3—C2—C11—O4	141.08 (11)
C1—C2—C3—C14	171.05 (10)	C11—O4—C12—C13	174.00 (14)
C14—C3—C4—C9	−138.81 (13)	C4—C3—C14—C15	−131.87 (13)
C2—C3—C4—C9	−16.27 (17)	C2—C3—C14—C15	106.22 (14)
C14—C3—C4—C5	47.28 (17)	C4—C3—C14—C19	48.91 (18)
C2—C3—C4—C5	169.83 (12)	C2—C3—C14—C19	−73.00 (16)
C9—C4—C5—O7	−170.20 (14)	C19—C14—C15—C16	−2.4 (2)
C3—C4—C5—O7	3.9 (2)	C3—C14—C15—C16	178.31 (13)
C9—C4—C5—C6	5.9 (2)	C14—C15—C16—C17	−0.2 (2)
C3—C4—C5—C6	179.98 (13)	C15—C16—C17—C18	2.5 (2)
O7—C5—C6—C7	−160.47 (15)	C15—C16—C17—N1	−175.77 (14)
C4—C5—C6—C7	23.4 (2)	O6—N1—C17—C16	−4.4 (2)
C5—C6—C7—C20	71.49 (18)	O5—N1—C17—C16	175.33 (16)
C5—C6—C7—C8	−49.37 (19)	O6—N1—C17—C18	177.29 (16)
C5—C6—C7—C21	−167.74 (15)	O5—N1—C17—C18	−2.9 (2)
C6—C7—C8—C9	48.98 (17)	C16—C17—C18—C19	−1.9 (2)
C20—C7—C8—C9	−72.17 (17)	N1—C17—C18—C19	176.34 (14)
C21—C7—C8—C9	167.47 (14)	C17—C18—C19—C14	−0.9 (2)
C5—C4—C9—O1	173.22 (13)	C15—C14—C19—C18	3.1 (2)
C3—C4—C9—O1	−0.7 (2)	C3—C14—C19—C18	−177.72 (14)

### Hydrogen-bond geometry (Å, º)

**Table:**

*D*—H···*A*	*D*—H	H···*A*	*D*···*A*	*D*—H···*A*
O2—H2···O3^i^	0.82 (2)	2.08 (2)	2.8881 (15)	172 (2)

Symmetry code: (i) *x*, −*y*+3/2, *z*+1/2.
